# Evaluation of 25-Hydroxyvitamin D Levels in Central Anatolia, Turkey

**DOI:** 10.1155/2018/4076548

**Published:** 2018-06-25

**Authors:** Ibrahim Solak, Fatma Goksin Cihan, Seher Mercan, Tugba Kethuda, Mehmet Ali Eryılmaz

**Affiliations:** ^1^Department of Family Medicine, Medical Sciences University, Training and Research Hospital, Konya, Turkey; ^2^Department of Family Medicine, Necmettin Erbakan University Medical Faculty, Konya, Turkey; ^3^Department of General Surgery, Medical Sciences University, Training and Research Hospital, Konya, Turkey

## Abstract

**Background/Aim:**

The aim of this study is to evaluate serum 25-hydroxyvitamin D levels measured within one year at an Education and Research Hospital in Turkey to identify vitamin D insufficiency/deficiency (which is very commonly seen in the society) and to provide a current approach to treatment management.

**Materials and Methods:**

This retrospective descriptive study was carried out by examining the data relating to 35,667 individuals.

**Results:**

Of the individuals participating in the study, 94.47% had a serum 25-hydroxyvitamin D level less than 30 ng/ml, 76.25% had a serum 25-hydroxyvitamin D level less than 20 ng/ml, and 32.20% had a serum 25-hydroxyvitamin D level less than 10 ng/ml. The mean serum 25-hydroxyvitamin D level of all individuals included in the study was 15.2±8.8 ng/ml. The mean serum 25-hydroxyvitamin D level was 14.5±8.8 ng/ml among women and 18.1±8.4 ng/ml among men, respectively (p<0.001).

**Conclusion:**

Vitamin D deficiency/insufficiency is widespread in the world and in Turkey. The data obtained from this study suggest that without measuring serum 25-hydroxyvitamin D level will be cost-effective in every age group (except for those are at risk of toxicity) as in children aged 0-1 year old in Turkey and that making a decision in this direction will make a great contribution to the economy of the country.

## 1. Introduction

Vitamin D is a fat-soluble vitamin that acts as a steroid hormone. Most of vitamin D is synthesized endogenously whereas a small amount of vitamin D is taken from foods. During exposure to sunlight, 7-dehydrocholesterol (a precursor of vitamin D) is synthesized in the skin. 7-dehydrocholesterol is hydroxylated in the liver to 25-hydroxyvitamin D. It is then hydroxylated in the kidney to 1,25-hydroxyvitamin D [1].

Sunlight exposure is required for vitamin D synthesis. The angle at which the sun's incident light hits the surface of the earth (Zenith angle) is effective in the synthesis of vitamin D. At latitude where Turkey is located, vitamin D synthesis occurs between May and November. Since the appropriate beam angle is obtained between 10.00-15.00 hours, it is recommended that people expose themselves to the sun between these hours for vitamin D synthesis [2].

The active form of vitamin D is 1,25(OH)_2_D_3_. 25-hydroxyvitamin D is the major circulating form of vitamin D. 25-hydroxyvitamin D is almost 1000-fold of that of 1,25(OH)_2_D_3_ [3]. While 25- hydroxyvitamin D has a half-life of 2–3 weeks, 1,25(OH)_2_D_3_ has only 4–6 hours half-life. For these reasons, it is the best known parameter to show the amount of vitamin D in the human body [4].

Vitamin D together with parathyroid hormone plays an important role in the regulation of calcium and phosphate homeostasis in the body. 1,25(OH)_2_D_3_ enhances calcium absorption in the duodenum and phosphate absorption in the ileum and reduces calcium excretion by the kidney [5].

Insufficient vitamin D production in the skin (which is caused by dark skin tones or inadequate exposure to sunlight) and inadequate dietary intake are the main reasons for low serum 25- hydroxyvitamin D levels [6]. Moreover, various diseases (intestinal malabsorption syndromes such as celiac and Crohn's disease) that inhibit the absorption of vitamin D are among the reasons for vitamin D deficiency [7].

Although there is no consensus on optimal levels of 25-hydroxyvitamin D, many researchers accept that 25(OH)D>30 ng/mL (75 nmol/L) is considered sufficient; vitamin D insufficiency is diagnosed when 25-hydroxyvitamin D is between 20 and 30 ng/mL (50-75 nmol/L); when 25(OH)D<20 ng/mL (50 nmol/L) it is vitamin D deficiency and if 25(OH)D<10 ng/mL (25 nmol/L) it demonstrates severe vitamin D deficiency. Vitamin D toxicity occurs when 25-hydroxyvitamin D levels are above 150 ng/mL [3, 8, 9].

It has been estimated that 1 billion people worldwide have vitamin D deficiency or insufficiency. When the literature is examined, vitamin D status differs among various countries and even in different regions of the same country. In Europe and the US, 40-100% of older women and men living in the society (not in care houses) have been reported to have vitamin D deficiency [10].

The aim of this study was to evaluate serum 25-hydroxyvitamin D levels measured within one-year period at an Education and Research Hospital in Turkey to identify vitamin D insufficiency/deficiency (which is very commonly seen in the society) and to provide a current approach to treatment and management.

## 2. Materials and Methods

This retrospective descriptive study was carried out by examining the data of 39,107 patients who were admitted to Health Sciences University Konya Education and Research Hospital for any reason between January 1, 2016 and December 31, 2016 and whose serum 25-hydroxyvitamin D levels were measured. Only the first admissions among the results obtained by the same identification number were taken into account. When repeated measurements were omitted, serum 25-hydroxyvitamin D levels of 35,667 participants remained in the study finally. The age and gender of the participants, date of admission, serum 25-hydroxyvitamin D level, and department that consulted the test were assessed. The city of Konya, where this study was conducted, is located at 37.87 North latitude. 25-hydroxyvitamin D level was measured using the enzyme-linked fluorescence assay (ELFA) with the “VIDAS® 25 OH Vitamin D Total” kit on the “Beckman Coulter Access 2 Immunoassay System” device in the same hospital's biochemistry laboratory. A serum 25-hydroxyvitamin D level higher than 30 ng/mL was considered sufficient, vitamin D insufficiency was defined as a serum 25-hydroxyvitamin D level between 20–30 ng/mL, and vitamin D deficiency was defined as a serum 25-hydroxyvitamin D level less than 20 ng/mL. The study protocol was approved by the ethics committee of Selcuk University (2017/1063).

### 2.1. Statistical Analysis

The conformity of the data to normal distribution was assessed with the Shapiro-Wilk test. The variables were expressed as mean ± standard deviation. A three-way ANOVA test was used to compare the independent variables. The Sidak test was performed for multiple comparisons. Analyses were performed using IBM SPSS Statistics software (version 20; IBM Corp., Chicago, IL, USA). A value of p < 0.05 was considered statistically significant.

## 3. Results

**Figure 1:**
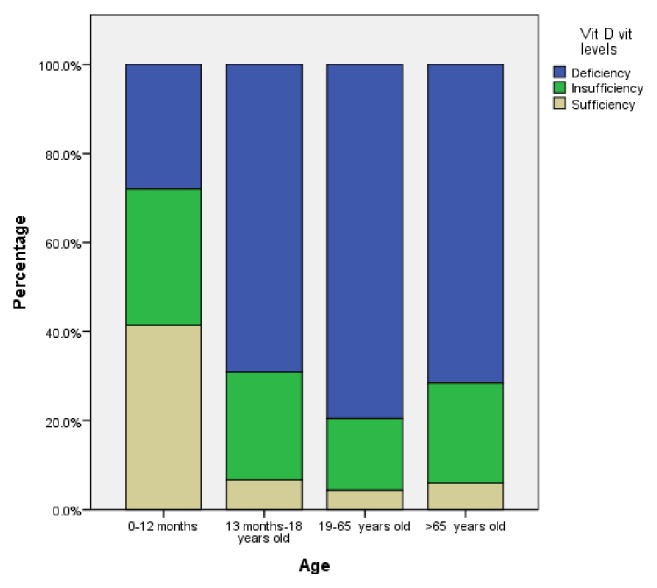
Distribution of vitamin D deficiency, insufficiency, and sufficiency according to age groups.

**Figure 2:**
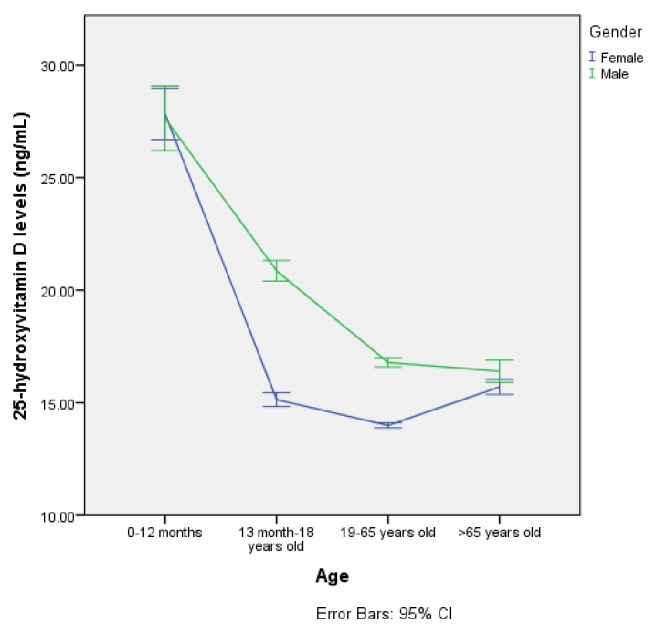
Comparison of serum 25-hydroxyvitamin D levels according to gender and age groups.

Of the patients participating in the study, 28,185 (79.0%) were females and 7482 (21.0%) were males. The mean age was 41.71±0.11 years for women and 37.99±0.266 years for men. There was a statistically significant difference between women and men in terms of age (p<0.001). When the ages of the participants were categorized into 0-12 months, 1-18 years, 19-65 years, and over 65 years, there was a statistically significant difference between the groups in terms of serum 25-hydroxyvitamin D level (p<0.001). The mean serum 25-hydroxyvitamin D levels according to gender and age groups are shown in Table 1. Of the participants, serum 25-hydroxyvitamin D levels of 94.47% were less than 30 ng/mL, 76.25% were less than 20 ng/mL, and 32.20% had serum 25-hydroxyvitamin D levels less than 10 ng/mL. The mean serum 25-hydroxyvitamin D level of all participants was 15.2±8.8 ng/mL. The serum 25-hydroxyvitamin D levels of the participants were found to be significantly deficient (<20 ng/mL) (p<0.001). The percentages for vitamin D deficiency, insufficiency, and sufficiency according to age groups are shown in Figure 1. The mean serum 25-hydroxyvitamin D level was 14.5±8.8 ng/mL among women and 18.1±8.4 ng/mL among men. Serum 25-hydroxyvitamin D levels were lower in women than in men (p<0.001). A comparison of serum 25-hydroxyvitamin D levels according to gender and age group is shown in Figure 2.

The 25-hydroxy vitamin D was screened during March and August (n:3565) at most and in May (n:2193) at least. Of the individuals included in the study, 11.132 (31.2%) were admitted to the physical medicine and rehabilitation outpatient clinic, 7597 (21.3%) to the internal medicine outpatient clinic, 3692 (10.4%) to the pediatric outpatient clinic, 3470 (9.7%) to the rheumatology outpatient clinic, 1923 (5.4%) to the endocrinology outpatient clinic, 1404 (3.9%) to the family medicine outpatient clinic, and the remaining patients to other various outpatient clinics.

**Figure 3:**
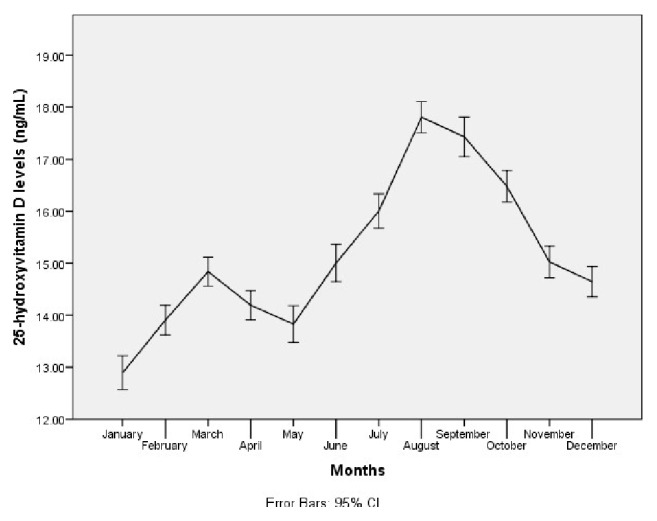
Serum 25-hydroxyvitamin D levels according to months.

**Figure 4:**
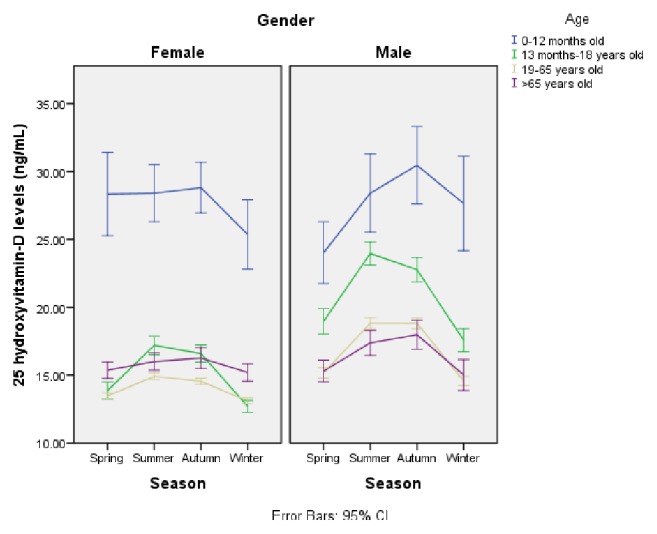
Comparison of serum 25-hydroxyvitamin D levels according to season, gender, and age group.

When the mean serum 25-hydroxyvitamin D levels were evaluated according to months, it was highest in August (17.8 ng/mL) and was lowest in January (12.9 ng/mL) (p<0.001). Serum 25-hydroxyvitamin D levels according to months are shown in Figure 3. Serum 25-hydroxyvitamin D levels were significantly lower in home care patients compared to other individuals (p<0.001). When the mean serum 25-hydroxyvitamin D levels were evaluated according to season, gender, and age group, the lowest value was detected during the winter season. A comparison of serum 25-hydroxyvitamin D levels according to season, gender, and age group is shown in Figure 4.

## 4. Discussion

Vitamin D deficiency/insufficiency is very common worldwide. Vitamin D deficiency is attracting greater attention among the academicians by the increasing number of scientific research studies and among the public by mentioning it very often in the printed and visual media and the Internet.

In a study which was conducted by Katrinaki et al. [11] in a large Mediterranean population of 8183 people, they found that the mean serum 25-hydroxyvitamin D level was lower among both women and men, in accordance with our study. Similar to our results, this study revealed that the mean serum 25 hydroxyvitamin D level was 19.4±9.5 (ng/mL+SD) among men and 18.01±9.01 (ng/mL+SD) among women. The mean serum 25-hydroxyvitamin D level was also lower in women than in men. In a multicenter study on 2173 apparently healthy adults who were recruited from 5 Chinese cities, the mean serum 25-hydroxyvitamin D level of the participants was 19.4±6.4 ng/mL. 25-Hydroxyvitamin D levels were lower in women than in men [12]. In a study conducted by Mansoor et al. [13] in Karachi region (Pakistan), they found that the mean serum vitamin D level was 16.4 ng/mL and that 69.9% had a serum 25-hydroxyvitamin D level less than 20ng/mL.

Turkey is one of the countries where vitamin D deficiency is endemic. In “Turkish Diabetes, Hypertension, Obesity and Endocrinological Diseases Prevalence Study II” (TURDEP-II), which is the most comprehensive survey in Turkey, 9560 adults living in rural and urban areas were examined and 93% had serum 25-hydroxyvitamin D levels less than 20 ng/mL [14]. In another study on people living in the city of Diyarbakir, which is located in the south east region of Turkey and has a hot and dry climate, when the cut-off value for vitamin D deficiency in the winter months was accepted as 20 ng/mL, the rate of vitamin D deficiency was 94% [15]. In our study, 76.25% had a serum 25-hydroxyvitamin D level less than 20 ng/ml. Consistent with other studies in the literature, the mean serum vitamin D level was lower in females than in males. In Turkey, the lack of vitamin D-fortified product usage contributes to insufficient oral dietary intake. The difference in serum 25-hydroxyvitamin D levels between genders may not be directly due to hormonal reasons. Most of the women in this study were housewives with sedentary lifestyle. An indoor sedentary lifestyle may affect sunlight exposure.

In a study performed by Santos et al. [16] in Portugal, serum 25-hydroxyvitamin D levels of the participants showed significant differences according to the seasons. The highest level was detected in summer, followed by autumn, spring, and winter. It was highest in September and was lowest in March. However, all of the serum 25-hydroxyvitamin D levels were under the cut-off value which is defined as vitamin D deficiency. Kroll et al. [17] reported that there was a seasonal variation observed in the United States. The change in blood levels of about 5 ng from winter to summer was consistent with their observation even though the levels were much lower. This probably reflects that some foods are fortified with vitamin D in the United States. In another study, it was highest in August and was lowest in January. The cut-off value for vitamin D deficiency was below 20 ng/mL even in August. It is expected that serum 25-hydroxyvitamin D levels are low due to less sun exposure in the winter months. Although sunlight exposure is considered to be related to the geographic allocation, the deficiency rate is about 39% in regions with intense sunlight, even in subtropical climate regions [18].

In our study, the highest 25-hydroxyvitamin D level was detected among infants aged 0-12 months. This can be explained by distributing free vitamin D supplements to infants aged 0-12 months in primary healthcare institutions in Turkey. In our study, the lowest 25-hydroxyvitamin D level was observed among adults aged 18–65 years. The majority of the members in this group are working in closed areas, and therefore they are exposed to less sunlight. The fact that serum 25-hydroxyvitamin D levels were higher in patients over 65 years old compared with patients aged 18-65 years old can be due to the combined use of calcium and vitamin D for osteoporosis in this group.

Regarding the high prevalence of vitamin D deficiency in the general population and the uncertainty about treatment of asymptomatic individuals leading to improved health outcomes, some authors question whether a large increase in serum tests leads to a potential increase in unnecessary healthcare costs [19–21].

The main strength of the present study was the large sample size. This study not only has the largest sample size to assess vitamin D status in Turkey but also is the second large sampled study in worldwide literature in English language [22]. Moreover, since our laboratory is one of the largest references laboratories in Turkey, the results are highly reliable.

The main limitation of this study was its retrospective design. The data were limited with the recorded information. We do not know the lifestyle conditions such as dietary habits, complaints, and treatment status of the participants. Nevertheless, there is a great fact that 94.47% had a serum 25-hydroxyvitamin D level less than 30ng/ml.

## 5. Conclusion

Vitamin D deficiency/insufficiency is widespread in the world and in Turkey. It is known that sunlight is not utilized enough due to various reasons today. In this context, the importance of vitamin D rich foods or vitamin D supplementation is increasing. With all of the mounting evidence for a wide variety of health benefits associated with sufficient vitamin D there is no downside to improving everyone's vitamin D status. The goal should be to maintain at least 30 ng/mL serum level of 25-hydroxyvitamin D; the preferred range is 40–60 ng/mL. This can be achieved by increasing vitamin D supplementation rates for everyone to reach the levels recommended by the Endocrine Society [10] as well as obtaining sensible sun exposure. In the light of our results and previous studies, it is clear that there is no need to screen everyone for the vitamin D status. It is much more cost-effective to increase food fortification with vitamin D and sensible sun exposure and encourage vitamin D supplementation much the same like the routine in 0-1 year old group. However those individuals with fat malabsorption syndromes and those who had gastric bypass surgery or have other risk factors or were born with acquired disorders in vitamin D metabolism do require screening [23–25].

## Figures and Tables

**Table 1:** Mean serum 25-hydroxyvitamin D levels (ng/mL) according to gender and age group.

Gender	Age group	n	Mean	SD
Female	0-12 months	352	27.8	10.9
	1-18 years	3000	15.1	8.6
	19-65 years	21712	13.9	8.5
	Over 65 years	3121	15.7	9.5
	Total	28185	14.5	8.8

Male	0-12 months	342	27.6	13.5
	1-18 years	1537	20.6	9.2
	19-65 years	4571	16.8	6.7
	Over 65 years	1032	16.4	8.1
	Total	7482	18.1	8.4

Total	0-12 months	694	27.7	12.3
	1-18 years	4537	17.1	9.2
	19-65 years	26283	14.5	8.3
	Over 65 years	4153	15.9	9.2
	Total	35667	15.2	8.8
